# Safeguarding of Fetal Growth by Mast Cells and Natural Killer Cells: Deficiency of One Is Counterbalanced by the Other

**DOI:** 10.3389/fimmu.2017.00711

**Published:** 2017-06-16

**Authors:** Nicole Meyer, Katja Woidacki, Marcus Maurer, Ana Claudia Zenclussen

**Affiliations:** ^1^Experimental Obstetrics and Gynecology, Medical Faculty, Otto-von-Guericke-University, Magdeburg, Germany; ^2^Charité, Universitätsmedizin Berlin, Berlin, Germany

**Keywords:** natural killer cells, mast cells, mast cell-specific proteases, spiral arteries, counter-regulation

## Abstract

Uterine natural killer cells (uNKs) and mast cells (uMCs) are of crucial importance for spiral artery (SA) remodeling and placentation. Mice deficient for both NKs and MCs including uNKs and uMCs show markedly impaired SA remodeling and their fetuses are growth-retarded. In contrast, the absence of either NKs or MCs results in only minor impairment. This suggests that uNKs can compensate for the effects of uMCs on SA remodeling and vice versa. To test this hypothesis, we assessed uNK numbers in uMC-deficient mice as well as uMC numbers in uNK-depleted mice. Notably, uMC-deficient C57BL/6J-Kit*^W-sh/W-sh^* (*W-sh*) mice showed markedly increased numbers of uNKs in contrast to wild type, and the transfer of bone marrow-derived MCs reverted this phenotype. Vice versa, uNK-deficient C57BL/6NTac-IL15*^tm1Imx^*N5 (IL-15^−/−^) mice had significantly increased numbers of uMCs and MC-specific proteases. Our results suggest that uNKs and uMCs can counterbalance their effects at the feto–maternal interface and jointly promote SA remodeling and placentation.

## Introduction

One milestone in pregnancy is the remodeling of spiral arteries (SAs) to adapt to the oxygen and nutrients demand from the growing fetus. During SA remodeling, the thick-walled arteries need to be transformed into thin-walled vessels so that they can fulfill the changing demand of blood from the growing fetoplacenta unit (1). By changing the shape of the SAs, the circulating blood volume can be increased by 10-fold, and the velocity of the maternal blood flowing into the intravillous space is reduced (2). This is important for a perfectly regulated exchange of nutrients, oxygen, and waste products and is, therefore, relevant for fetal development.

Uterine natural killer cells (uNKs) are the most abundant lymphocyte population at the feto–maternal interface of humans and mice (3, 4). Mating of NK-deficient, C57BL/6NTac-IL15*^tm1Imx^*N5 [interleukin (IL)-15^−/−^] animals (5) demonstrated that the absence of uNKs negatively affects decidual transformations at the feto–maternal interface during early pregnancy and results in impaired SA remodeling (6, 7). It was long believed that uNKs are the only cells in charge of SA remodeling. However, we recently reported that uterine mast cells (uMCs) also contribute to SA remodeling (8) using mast cell (MC)-deficient C57BL/6J-Kit*^W-sh/W-sh^* (*W-sh*) mice as a model (8, 9). MC deficiency in these mice is based on an inversion mutation in the *white spotting* (*W*) locus leading to a restricted c-Kit (CD117) tyrosine-dependent signaling. An advantage of the model is the possibility to reconstitute the mice systemically or locally with bone marrow-derived MCs (BMMCs) to confirm that the phenotype is due to the absence of MCs *in vivo* (9). Indeed, reconstitution of *W-sh* mice with BMMCs fully restored the reproductive phenotype, confirming the participation of MCs in SA remodeling.

To better understand the contribution and relevance of uNKs and uMCs in the regulation of SA remodeling, we recently established a model in which both MCs and NKs are missing. For this, we employed carboxypeptidase (Cpa)3 mice constitutively lacking MCs and depleted NKs by using an antibody approach (10). In these uMC/uNK-deficient mice, we observed nearly un-remodeled SAs and drastic effects on pregnancy outcome and the progeny, with more than half of the fetuses affected by intrauterine growth restriction (IUGR) (10). This was unexpected as mice that lack uMCs (but have uNKs) and mice that lack uNKs (but have uMCs) only show minor, albeit distinct, impairment of SA remodeling and largely normal pregnancy outcome. We, therefore, hypothesized that uNKs can compensate for the absence of uMCs and their effects on SA remodeling and vice versa. To test this hypothesis, we analyzed the numbers and distribution of uNKs in MC-deficient *W-sh* mice and Cpa3^Cre/+^ mice. Additionally, we determined the frequency and distribution of uMCs in uNK-deficient IL-15^−/−^ mice. In both models, SA remodeling and pregnancy outcome were studied.

## Materials and Methods

### Animal Models and Experimental Setup

Female C57BL/6J and C57BL/6N mice as well as male BALB/c were obtained from Charles River (Sulzfeld, Germany). MC-deficient female C57BL/6J-Kit*^W-sh/W-sh^* (*W-sh*) mice were bred and maintained at Charité in Berlin, Germany, and then transferred to Magdeburg 2 weeks prior to the experiments. Female IL-15-deficient C57BL/6NTac-IL15*^tm1Imx^*N5 were purchased from Taconic (New York, NY, USA) and bred in our facilities. MC-deficient C57BL/6J-Cpa3^Cre/+^ (Cpa3^Cre/+^) mice and their controls C57BL/6J-Cpa3^+/+^ (Cpa3^+/+^) were kindly provided by Prof. H. R. Rodewald (Heidelberg, Germany) and bred in our facilities. Mice were housed in our barrier facility with a 12-h light/dark cycle (7:00 a.m.–7:00 p.m./7:00 p.m.–7:00 a.m.) and received food and water *ad libitum*. Experiments were performed according to the institutional guidelines upon ministerial approval (42502-2-1296UniMD). 6–8 weeks old females were paired with BALB/c males, checked twice a day for vaginal plugs, and separated from the males if mated. Plug appearance was defined as gestation day (gd) 0.

Female *W-sh*, Cpa3^Cre/+^ and control mice were sacrificed at gd10, and one implantation site from the end of the left uterus horn was removed for the evaluation of uNK numbers. C57BL/6N and IL-15^−/−^ mice were sacrificed on gd5, gd10, or gd18. For the group sacrificed at gd5, uterus samples were removed for histological, flow cytometry, and RNA analysis of uMCs. On gd10, blood was taken for flow cytometry analysis, and implantation sites were documented and removed for quantification of uNKs and SA analysis. Spleen, mesenteric, and inguinal lymph modes (LNs) were removed for flow cytometry analysis. One decidua piece was snap frozen for later RNA analysis. A further group was employed for blood pressure measurements and sacrificed at gd18; thereafter, fetal and placental weights were measured and the fetus-to-placenta weight ratio index (FPI) was calculated.

### Sample Collection

Mice were anesthetized, and blood was obtained by retroorbital puncture; afterward, they were sacrificed and the implantation rate was determined by registering the total number of fetuses. Spleen, mesenteric LNs, and inguinal LNs were removed, washed in cold phosphate-buffered saline (PBS), and conserved in cold RPMI until use for flow cytometry analysis. For histological analysis, two implantations per female were removed, fixed in 4% paraformaldehyde (PFA) containing 0.1 M saccharose (pH 7.4) for 6 h at room temperature (RT), and embedded in paraffin after dehydration with ethanol and xylol cycles. One piece of the uterus (gd5) or decidua (gd10) was cut into small pieces and stored in RPMI supplemented with 5% penicillin–streptomycin (P/S) containing 0.005% liberase for flow cytometry. A further piece was fixed in ethanol (96%) at 4°C and later embedded in paraffin after dehydration with ethanol and xylol cycles. Uterus (gd5) or decidua (gd10) tissue was additionally collected for RNA isolation, washed in cold PBS, snap-frozen in liquid nitrogen, and stored at −80°C until use.

### Isolation, Culture, and Transfer of BMMCs

Isolation, culture, and transfer of BMMCs were performed as previously described (8). Shortly, wild-type (WT) bone marrow cells from femur and tibia were cultured in Iscove’s modified Dulbecco’s medium supplemented with 10% fetal bovine serum (FBS), 1% P/S, and murine recombinant IL-3 (30 ng/ml, Life technologies, Carlsbad, CA, USA). After confirmation by flow cytometry analysis that >95% of the cells were CD117^+^FcεRIα^+^ BMMCs, they were used for reconstitution. BMMCs were transferred into 10-week-old *W-sh* females by i. v. injection of 5 × 10^6^ cells in 200 µl of PBS in the morning of two consecutive days (total 1 × 10^7^ cells). 12 weeks after reconstitution, *W-sh* females were mated with BALB/c males (8).

### Dolichos Biflorus Agglutinin (DBA) Lectin Histochemistry

To compare uNK numbers between the experimental groups, implantation sections were stained with DBA lectin. After dewaxing and rehydration procedure, endogenous peroxidases were blocked by incubation with 3% hydrogen peroxide (H_2_O_2_) in methanol at RT for 30 min in a humidity chamber. Slides were washed in 50 mM PBS twice, followed by 15 min avidin and biotin blocking each. Prior to DBA lectin staining [dilution 1:150 in 1% bovine serum albumin (BSA) in 100 mM PBS, overnight (ON) at 4°C], proteins were blocked with 1% BSA in 100 mM PBS for 30 min. For negative controls, DBA lection staining solution was replaced by 1% BSA in 100 mM PBS. At the next day, sections were washed twice with PBS and treated with horseradish peroxidase-solution for 30 min. After staining, slides were washed twice and incubated with AEC substrate chromogen (Dako) for 6 min and washed twice again. Sections were counter-stainined with hematoxylin solution for 1–2 min, dipping in warm tap water and mounted with Aqua Tex. uNKs were identified as DBA lectin reactive, brown cells, and their number was calculated per 1 mm^2^ with the help of an eyepiece-micrometer (Zeiss) and a light microscope (Zeiss).

### Flow Cytometry Analysis and Antibodies

Flow cytometry was used to compare percentages of peripheral (p) NKs or uMCs among the experimental groups. Single cell suspensions from uterus (gd5) or decidua (gd10) samples were obtained by enzymatic digestion with RPMI containing 5% P/S and 0.005% liberase for 90 min in the CO_2_ incubator (37°C, 5% CO_2_). Afterward, liberase was inactivated with RPMI supplemented with 10% FBS and 1% P/S, the tissue was squashed and filtered (100 µm net) and incubated further 30 min with RPMI + 10% FBS + 1% P/S in the CO_2_ incubator. After centrifugation, the pellet was resuspended in 100 µl FACS buffer (10 g BSA, 1 g NaN_3_ added to 1 l with PBS). Besides uterus/decidua samples, spleen, blood, mesenteric, and inguinal LNs were isolated, crushed, and filtered (100 µm net). Afterwards, erythrocytes were lysed with lysis buffer [89 g NH_4_CL, 10 g KHCO_3_, 0.38 g EDTA add to 1 l with aqua dest for preparing lysis buffer (10×)] for 10 min. Washing with RPMI followed centrifugation (1,200 rpm, 10 min, 4°C), than single cells suspensions were resuspended in 100 µl FACS buffer. Suspensions were stained 30 min in the darkness at 4°C with the following antibody solutions (dilution 1:100 with FACS buffer): FITC-conjugated rat anti-mouse CD122 (clone: 5H4), PerCP-conjugated hamster anti-mouse CD3 (clone: 145-2C11), and PE-conjugated mouse anti-mouse NK1.1 (clone: PK136) have been utilized for analysis of pNKs. PE-conjugated rat anti-mouse CD117 (clone: 2B8) and FITC-conjugated hamster anti-mouse FcεRα (clone: MAR-1) for examination of uMCs. FcεRα antibody was purchased from eBioscience (Frankfurt, Germany), all other antibodies were purchased from BD Pharmingen (Heidelberg, Germany). Unstained cells or cells stained with isotype antibodies served as controls. Cells were washed, resuspended with 100 µl FACS buffer, and measurement with FACS Calibur (BD Bioscience) after staining. Data analysis with CellQuest Pro software (BD Bioscience) and statistical analysis with GraphPad Prism 5.0 followed.

### Analysis of SA Parameters

Spiral artery wall and lumen perimeter of two to ten SAs per implantation per female (gd10) were measured in hematoxylin-eosin (H/E)-stained implantation sections using a light microscope (magnification ×200) and the AxioVision 4 software (Zeiss, Germany). Calculation of SA wall and lumen diameter and determination of wall-to-lumen ratios followed. Afterward, the mean of wall to lumen ratio of multiple SA per one female was employed.

### H/E Staining

After standard dewaxing and rehydration procedure, implantation sections on slides were stained for 2 min with hematoxylin, rinsed twice in warm tap water, stained 1 min with eosin, and finally rinsed in distilled water. Dehydration by dipping the slides 10 times in each ethanol concentration (75, 95, 100%) and incubation in xylene for 2 min twice followed. Roti-Histokitt was used for covering the slides.

### Immunofluorescence

After fixation in 4% PFA and ethanol, one implantation per female was embedded in paraffin and cut in 5 µm sections. Immunofluorescence staining was performed after standard dewaxing and rehydration procedure. Staining required antigen retrieval treatment that was carried out in 0.1 M citrate buffer (pH 6.0) for 10 min in the microwave. Afterward, samples were washed and incubated at 4°C ON with anti-smooth muscle (SM) actin (clone: 1A4, Dako, dilution 1:50 in 10% BSA in TBS), followed by washing and incubation with the Alexa Fluor 555-conjugated secondary antibody goat anti-mouse (Life technologies, dilution 1:500 in 10% BSA in TBS) for 2 h at RT. For nuclear counterstaining VECTASHIELD^®^ mounting medium with DAPI (VECTOR laboratories) was used.

### Blood Pressure Measurement

To investigate if insufficient SA remodeling influences blood pressure in pregnant mice, systolic and diastolic blood pressure was determined in female mice by non-invasive tail cuff method using the TSE Blood Pressure Monitoring Systems (TSE Systems) and calculating the mean of 10 single values. Before starting the measurements, mice were located under an infrared lamp for 10 min. A training period of 2 weeks preceded the experiments. Blood pressure was monitored every second day from gd0 to gd18.

### Weight Determination

Individual fetal and placental weights were measured at gd18 using a micro scale (Kern & Sohn GmbH), with FPI (fetoplacental index) calculated from these values.

### Toluidine Blue Staining

In order to visualize uMCs as purple cells with granula, uterine sections from gd5 were stained with toluidine blue. After standard dewaxing and rehydration procedures, slides were incubated for 45 s in 0.1% toluidine blue solution and rinsed briefly in distilled water. Slides were dehydrated by dipping them 10 times in ethanol 75, 95, and 100% and incubation in xylene twice for 2 min each afterward. Slides were covered with Roti-Histokitt.

### RNA Isolation, c-DNA Synthesis and Quantitative Real-time PCR

Total RNA was extracted from frozen uterus tissue (gd5) and frozen decidual tissue (gd10) using TRIzol^®^ (Life technologies) reagent. Tissue was homogenized using an Ultra Turrax T25 homogenizer (NeoLab) while adding TRIzol^®^. RNA extraction with chloroform, precipitation with isopropanol, and washing with ethanol followed. RNA was diluted in RNA-free water, quantification was performed by ultraviolet absorbance at 260 nm, quality check by measuring absorbance at 260/280 nm (Synergy HT, Bio Tek Instruments). RNA storage occurred at −80°C. The RNA was reversed transcribed as followed: 2 µg of total RNA were incubated with oligo dTs and RNA-free water 10 min at 75°C and rested 2 min on ice. Incubation for 30 min at 37°C with dNTPs (2.5 mmol/l), DNase I (2 U/μl), and RNase inhibitor (40 U/μl) in a reaction buffer followed. Afterward, DNAse inactivation for 5 min at 75°C took place. After resting on ice for 2 min, reverse transcriptase (200 U/μl) was added together with RNase inhibitor (40 U/μl) and cDNA-synthesis occurred at 42°C for 60 min. Samples were stored at −20°C subsequent to inactivation of reverse transcriptase for 5 min at 94°C. Real-time polymerase chain reaction amplifications were performed with SYBR Green (Applied Biosystems) and an iQ5 Multicolor Real-Time PCR Detection System (BioRad). Each amplification reactions consisted of 1 µl cDNA, 6.5 µl SYBR Green PCR mastermix, 2 µl RNAse free water, 3 µl primermix, and 0.5 µl fluoresceine (50 nM). No template controls contained water instead of cDNA. The following primer were used: *Mcpt1* (fwd: TGG CAC TTC TCT TGC CTT CT; rev: GCT CAC ATC ATG AGC TCC AA), *Mcpt6* (fwd: GAC ATC GTG CTG GGT GAC; rev: ATA AGG AGG TGG GAG AGG C), and normalized to the housekeeping *β-Actin* (fwd: TGC GTC TGG ACC TGG CTG G; rev: ATC CTG TCA GCA ATG CCT GGG). Reactions were performed in duplicates as follows: 10 min 95°C, followed by 40 cycles of 30 s 95°C, 45 s 60°C for *Mcpt1* or 45 s 55°C for *Mcpt6*, 30 s 72°C, and finally once 1 min 95°C.

### Statistical Analysis

Experimental results were analyzed with GraphPadPrism5 (GraphPad software). Numbers of animals/samples, statistical test, and *P-*values are indicated in each figure legend. *P-*values of <0.05 were considered to be statistically significant. Normality of the data was first assessed using the D’Agostino Pearson-Omnibus test before analyzing the data using parametric or non-parametric tests. Depending on their distribution, data are presented as medians or means ± SEM. Implantation rates and data obtained by flow cytometry or real-time PCR were analyzed with the Mann–Whitney *U*-test. For blood pressure values, histological measurements, fetal weights, placental weights, and FPI unpaired *t*-test were used for analysis of differences between the animal groups.

## Results

### Mast Cells Critically Regulate uNK Numbers

**Figure 1:**
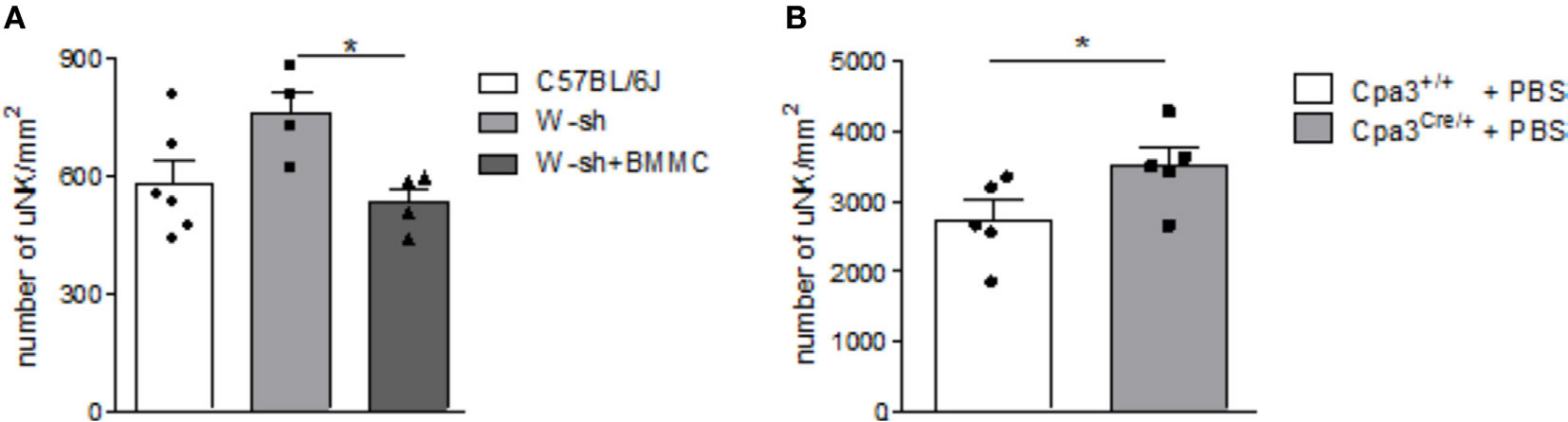
Mast cell (MC)-deficient mice show increased numbers of uterine natural killer cells (uNKs) in contrast to wild type (WT). Bone marrow-derived MCs (BMMC) reconstitution restored this phenotype. Numbers of uNKs cells per 1 mm^2^ were quantified in *Dolichos biflorus agglutinin* lectin-stained 5 µm paraffin cross sections of implantations of WT C57BL/6J (*n* = 6), MC-deficient *W-sh* (*n* = 4), or BMMC-reconstituted *W-sh* (*n* = 4) mice **(A)** as well as in Cpa3^+/+^ control mice (*n* = 5) vs. MC-deficient Cpa3^Cre/+^ (*n* = 5) mice **(B)** at gd10. Results are presented as means with SEM. Also individual values are shown by dots, squares and triangles. Statistical differences were obtained using Mann–Whitney *U*-test (*P < 0.05). MCs, mast cells; NKs, natural killer cells; u, uterine; WT, wild type; BMMC, bone marrow-derived mast cells; *W-sh*, C57BL/6J-Kit*^W-sh/W-sh^*.

Numbers of uNKs were markedly, albeit not significantly, increased in MC-deficient *W-sh* mice compared to C57BL/6J mice (*P* = 0.0656) as assessed by staining of implantation cross sections obtained at gestation day (gd) 10 stained for DBA lectin (Figure 1A). Reconstitution of *W-sh* mice with BMMCs resulted in a significant reduction of uNKs and normalized uNK numbers (Figure 1A). uNK numbers were also significantly increased in MC-deficient Cpa3^Cre/+^ mice compared to WT Cpa3^+/+^ mice (Figure 1B).

### NK Cell Absence in Pregnant Females Was Associated with Suboptimal SA Remodeling but Had No Effect on Blood Pressure Levels throughout Pregnancy

**Figure 2:**
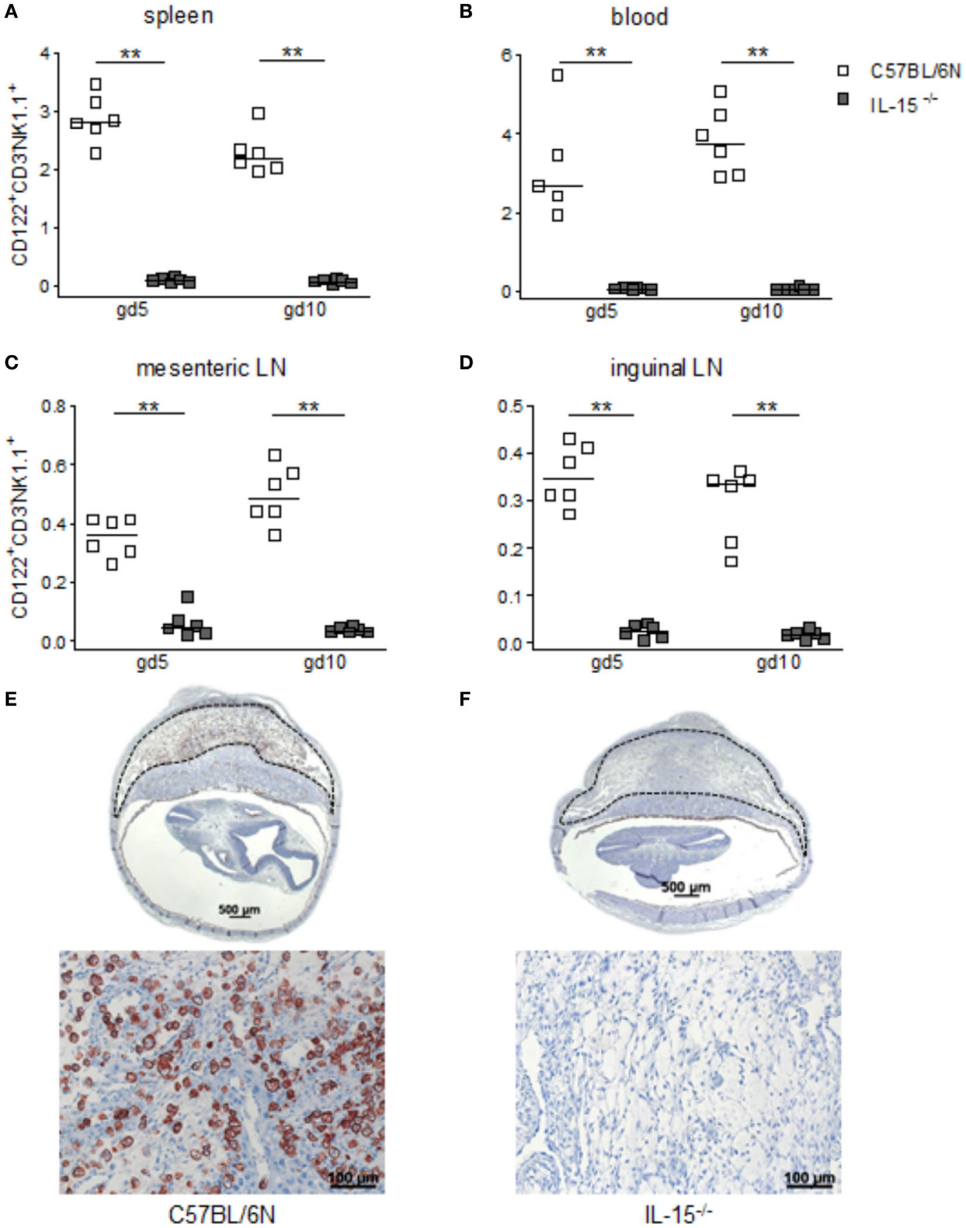
Interleukin (IL)-15-deficient mice are characterized by very low levels of peripheral (p) and the absence of uterine natural killer cells (uNKs) in contrast to wild-type mice. Percentages of CD122^+^CD3^−^NK1.1^+^ pNKs in spleen **(A)**, blood **(B)**, mesenteric lymph nodes (LN) **(C)**, and inguinal LNs **(D)** at gd5 and gd10 were analyzed by flow cytometry in samples from C57BL/6N (*n* = 5–6) or IL-15^−/−^ (*n* = 6) mice. Results are presented with squares showing the individual values for each mouse and medians are indicated. Statistical analysis was performed by the Mann–Whitney *U*-test (**P < 0.001). Representative Dolichos biflorus agglutinin (DBA) lectin-stained cross sections of implantations from C57BL/6N **(E)** or IL-15^−/−^ **(F)** mice at gd10 are depicted (scale bar = 500 μm). uNKs can be identified as DBA lectin-positive, brown-stained cells within the *decidua basalis* (dashed lines marked area). Lower pictures show cutouts from the *decidua basalis* with higher magnification (scale bar = 100 μm) **(F)** confirms the absolute NK absence at gd10. IL, interleukin; p, peripheral; u, uterine; LN, lymph nodes; DBA, dolichos biflorus agglutinin.

**Figure 3:**
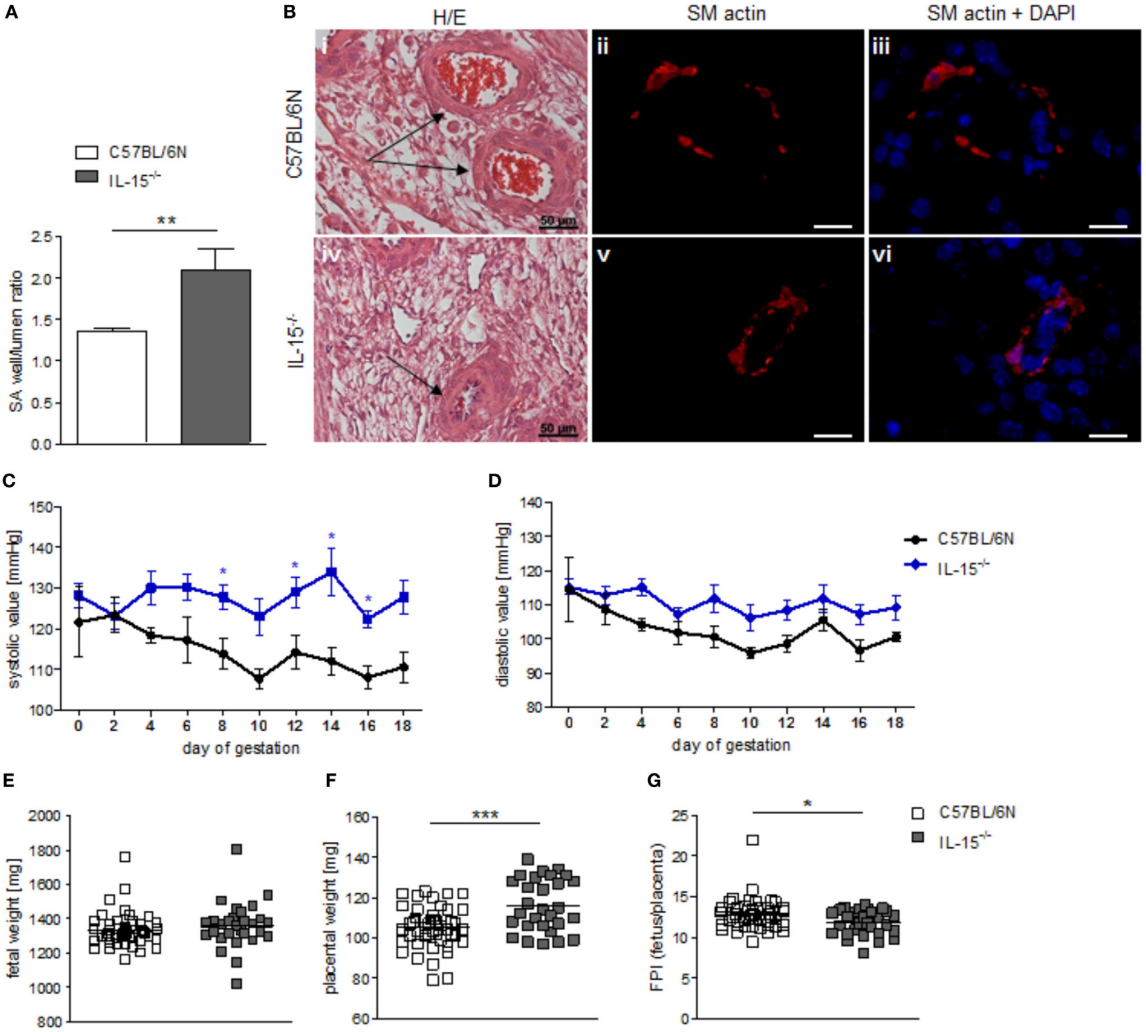
Interleukin (IL)-15^−/−^ mice showed impaired remodeling of spiral arteries (SAs), but no hypertension throughout pregnancy. While natural killer cell (NK) absence had no impact on fetal growth, it was associated with higher placental weight and lower fetoplacental-index (FPI). **(A)** SA wall and lumen perimeters of 3–10 SAs per animal of BL/6N (*n* = 6) and IL-15^−/−^ (*n* = 5) mice were measured and SA wall-to-lumen ratio were calculated. Results are presented as means with SEM. Statistical differences were obtained using Mann–Whitney *U*-test (**P < 0.01). **(B)** Representative images of H/E-stained sections (scale bar = 50 μm) and immunofluorescence for smooth muscle (SM) actin and SM actin + DAPI (scale bar = 20 μm) of SAs (indicated by arrows) of BL/6N and IL-15^−/−^ mice are shown. Systolic **(C)** and diastolic blood **(D)** pressure values for pregnant C57BL/6N (*n* = 5) or IL-15^−/−^ (*n* = 5) mice from gd0 to gd18. Data were recorded every second day. For **(C,D)**, results are presented as mean ± SEM. Statistical differences were obtained using Mann–Whitney *U*-test (*P < 0.05,). Fetal weights **(E)**, placental weights **(F)**, and FPI **(G)** from C57BL/6N (mothers: *n* = 5, fetus/placenta: *n* = 46) or IL-15^−/−^ (mothers: *n* = 5, fetus/placenta: *n* = 29) females at gd18. For **(E–G)**, results are presented with squares showing individual values for each fetus/placenta and means are indicated. Statistical differences were obtained using unpaired *t*-test (*P < 0.05, ***P < 0.001). IL, interleukin; SA, spiral arteries, NK, natural killer cells; FPI, fetoplacental-index; SM, smooth muscle.

When we used IL-15^−/−^ mice to assess the effects of NK cells on MCs, we first confirmed that these mice are NK deficient by flow cytometry and immunohistochemistry. Indeed, IL-15^−/−^ females exhibited dramatically decreased numbers of CD122^+^CD3^-^NK1.1^+^ NKs at gd10 in the spleen, blood, as well as mesenteric and inguinal lymph nodes (Figures 2A–D). Their uNK count was near 0 (Figures 2E,F). We next studied whether NK absence influences the number of implantations or SA remodeling. Pregnant IL-15^−/−^ and C57BL/6N control mice showed comparable numbers of implantations (data not shown). As indicated by a statistically significant increased wall-to-lumen ratio (Figure 3A) at gd10 (*P* < 0.01), SA remodeling was impaired in IL-15^−/−^ females. Animals lacking NKs showed SA with a striking small lumen diameter and thicker arterial wall (Figure 3B iv) in contrast to WTs (Figure 3B i). Additionally, there was an increased expression of SM actin in vascular smooth muscle cells of IL-15^−/−^ mice (Figure 3B v, vi) in contrast to WT animals (Figure 3B ii, iii). Staining for SM actin helps visualizing the thickness of SM layers that should have been removed during an effective SA remodeling process, with a thicker SM layer indicating insufficient SA remodeling. Defects in SA remodeling are often causative of maternal hypertension in humans (2, 11, 12). We, therefore, investigated next whether the impaired SA remodeling in IL-15^−/−^ mice is associated with maternal hypertension. Using a tail-cuff system, we monitored systolic (Figure 3C) and diastolic (Figure 3D) maternal blood pressure throughout pregnancy and observed a statistically significant increase in systolic blood pressure at gd8, 12, 14, and 16 in IL-15-deficient mice in contrast to C57BL/6N mice. Nevertheless, none of the animals developed hypertension (>140 mmHg) at any pregnancy time point. To evaluate a possible influence of IL-15 deficiency on fetal development, weight of fetuses and placentas was measured and FPI was determined. At gd18, there were no differences in fetal weight (Figure 3E) between the groups. Interestingly, there was a statistically significant increase in placental weight (Figure 3F) in IL-15^−/−^ mothers in contrast to C57BL/6N controls (*P* < 0.001). Based on this, a statistically significant lower FPI (Figure 3G), indicative of an insufficient placental development, resulted in IL-15^−/−^ animals in contrast to the WT group (*P* < 0.05).

**Figure 4:**
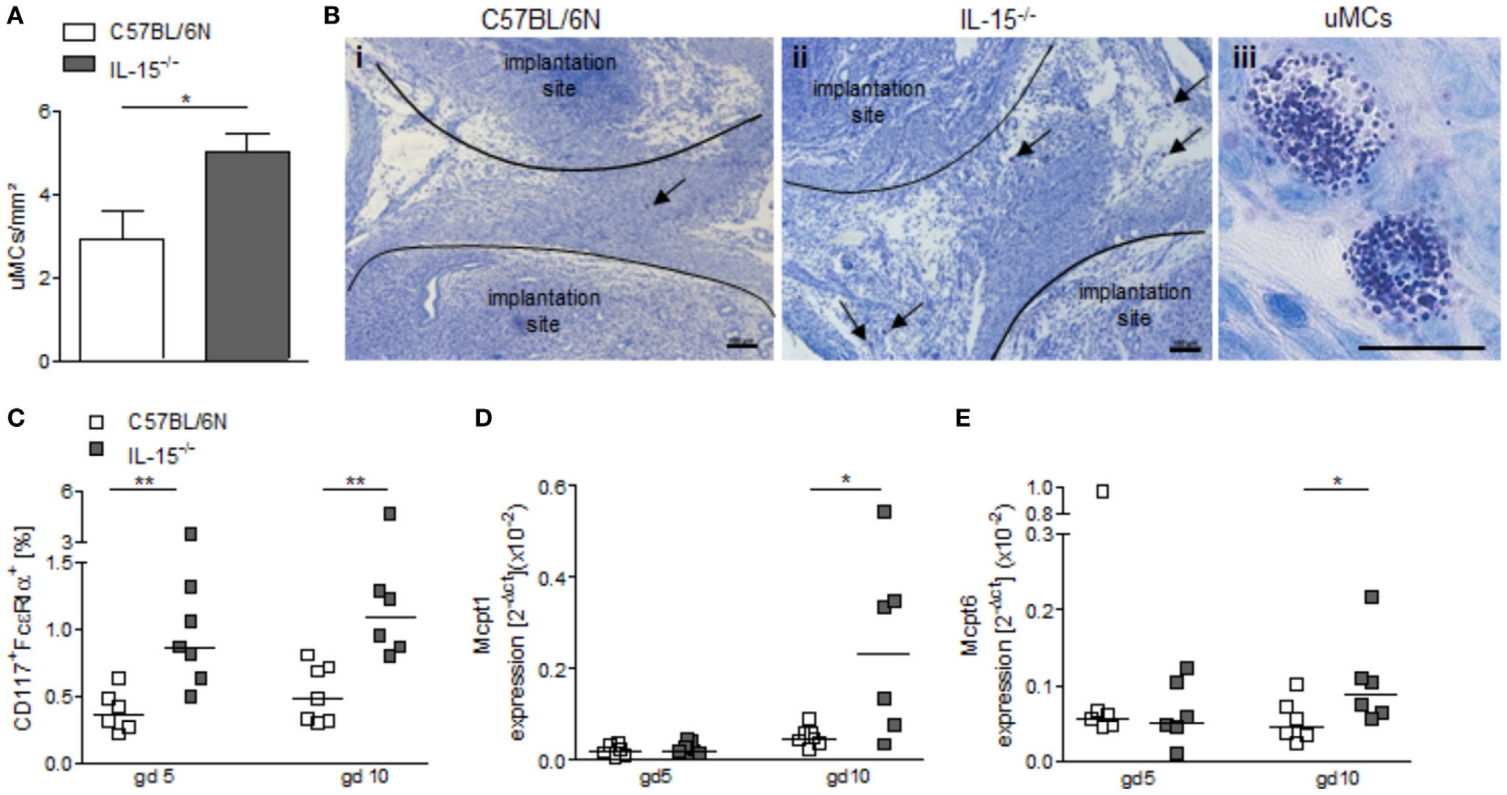
Interleukin (IL)-15^−/−^ mice exhibit increased numbers of uterine mast cells (uMCs). **(A)** Numbers of uMCs per 1 mm^2^ were quantified in tolouidine blue-stained 5 µm paraffin sections from uteri of C57BL/6N (*n* = 6) and IL-15^−/−^ mice (*n* = 6) at gd5. Results are presented as mean with SEM. Statistical differences were obtained using Mann–Whitney *U*-test (*P < 0.05). **(B)** Representative pictures for **(A)** are shown for C57BL/6N (i) and IL-15^−/−^ (ii) mice (scale bar = 100 μm). uMCs (iii) are localized between the implantation sites and are indicated by arrows (scale bar = 20 μm) in (i) and (ii). **(C)** Percentages of CD117^+^FcεRα^+^ MCs in uterus (gd 5) or decidua (gd 10) samples were analyzed by flow cytometry. mRNA levels of MC-related proteases (Mcpt)-1 **(D)** and Mcpt-6 **(E)** in uterine (gd 5) and decidual (gd 10) tissue of BL/6N and IL-15^−/−^ mice were measured. For **(C–E)**, results are presented with squares showing individual values. Additionally, the medians are indicated. Statistical differences were obtained using non-parametric Mann–Whitney *U*-test (*P < 0.05, **P < 0.005). IL, interleukin; uMC, uterine mast cell; Mcpt, MC-related protease.

NK-deficient IL-15^−/−^ mice exhibited significantly higher numbers of uMCs compared to WT as assessed by toluidine blue staining of gd5 uterus cross sections (Figures 4A,B, *P* < 0.05). Flow cytometry analyses of uterine tissue from animals at gd5 as well as of decidual tissue from animals at gd10 confirmed this and showed markedly increased rates of CD117^+^FcεRα^+^ MCs in uNK-deficient mice as compared to WT (Figure 4C, *P* < 0.01). Furthermore, real-time PCR analyses demonstrated that gd10 decidua samples of uNK-deficient females have a statistically significantly higher expression of the MC mediators Mcpt1 and Mcpt6 as compared to WT (Figures 4D,E, *P* < 0.05). In contrast, uterus samples from gd5 showed no differences in the expression of Mcpt1 and Mcpt6 (Figures 4D,E). Collectively, these findings indicate that uMCs and MC-related mediators are upregulated in NK-deficient mice.

## Discussion

Adaption of the maternal immune system during pregnancy is an absolute requirement for an unrestricted fetal development. In this regard, the finely modulated interplay of maternal immune cells is of enormous significance. Cells of the innate immune system contribute to early pregnancy by preparing the uterine tissue for implantation (13). The most studied cells in this context are uNKs. They are reportedly involved in the remodeling of SA by secreting IFN-γ (14). Additionally, our previous studies proved an important role for uMCs in reproductive processes. We showed that MC-deficient *W-sh* mice exhibited impaired remodeled SAs and a reduced placental size in contrast to MC-sufficient controls that can be reverted by previous MC reconstitution *via* BMMC transfer (8). By using mice that are devoid of both uNKs and uMCs, we could recently show that they jointly work to ensure a proper SA remodeling; their joint absence provoked nearly un-remodeled SA with a strong IUGR phenotype, with more than half of the fetuses being growth-retarded (10). As this phenotype was not as evident in females lacking either uNKs or uMCs, we propose that these cells have redundant functions so to ensure this pregnancy-relevant process. We also hypothesize that the absence of one cell type will lead to the augmentation in number or function of the other one.

Here, histochemical studies showed an increased number of uNKs in decidual tissue of MC-deficient *W-sh* mice. Furthermore, the reconstitution of the MC pool after BMMC transfer normalized uNK number. We additionally found higher numbers of uNKs in Cpa3^Cre/+^ MC-deficient mice, confirming an uNK augmentation if MCs are absent that is not model-dependent but rather characteristic for MC deficiency. It is, therefore, tempting to speculate that the uNK increase is directly related to the absence of uMCs. As both have the same function, at least regarding SA remodeling, we propose that these cells counter-regulate each other at the feto–maternal interface. To prove this hypothesis, we moved to NK-deficient IL-15^−/−^ mice (5) that had a significant reduced number of pNKs in blood, spleen, mesenteric, and inguinal LNs when compared to the controls. Additionally, we can affirm the complete absence of DBA lectin ^+^ uNKs in IL-15^−/−^ mice at day 10 of pregnancy.

Uterine natural killer cells accumulate in high numbers in the uterus during early gestation (15, 16), being the most abundant lymphocyte population at the feto–maternal interface (3, 4). They participate in several reproductive processes and their absence leads to impaired placental development and suboptimal SA remodeling albeit not affecting fetal growth (6, 7, 14). Here, we indeed confirmed that, at gd10, SAs of IL-15^−/−^ females showed remarkable decreased lumen diameters and thicker walls in H/E-stained implantation sections, which resulted in higher wall-to-lumen ratios in contrast to WT animals. Kieckbusch and colleges recently proposed a MHC-dependent inhibition of uNKs as causative of insufficient remodeling of decidual arteries. As the remodeling of SA guarantees the exchange of nutrients, oxygen, and waste products between mother and fetus, it was long believed that fetuses from NK-deficient mothers will be not able to survive *in vivo* and may die intrauterine or be born growth retarded. Surprisingly, animals born to mothers devoid of NKs are not growth restricted despite their insufficient remodeling (7), suggesting compensatory mechanisms to avoid fetal stress.

Insufficient SA remodeling in human is often associated with hypertensive disorders or development of small for gestational age progeny (2, 12, 17). We observed in our study that IL-15-deficient females had a significant higher systolic blood pressure from gd4 until the end of pregnancy in contrast to WT controls. However, none of the animals developed hypertension (>140 mmHg) at any pregnancy time point. Similarly, no direct relationship between efficiency of SA remodeling and changes in blood pressure or fetal size could be found when pregnant Rag2^−/−^/Il2rg^−/−^ mice were compared tom BALB/c mice (18). This, again, suggests either NKs being an associated marker but not the sole cause of inappropriate SA remodeling or the existence of compensatory mechanisms to impede hypertension and so to avoid fetuses to grow inappropriately. In the same line, Barber and Pollert documented a normal gestation period, comparable progeny yield, and comparable fetal weight to control mice when studying IL-15^−/−^ animals (7). While the values for fetal weight were comparable among the groups in our study, placental weight values were significantly increased in IL-15^−/−^ mice at gd18 in contrast to WT controls. Calculating the FPI from fetal and placental weights is usually employed as an indirect measurement of placental insufficiency (19). IL-15^−/−^ mice showed a significant lower FPI in contrast to the control group, a fact that associates IL-15 deficiency with placenta insufficiency. The link between NKs and placental development has already been proposed in other studies (20, 21). Heavier placentas in the IL-15^−/−^ group may represent a compensation system to augment the amount of blood flowing into the fetoplacental unit in order to compensate impaired SA remodeling. Collectively, our results and published data from other groups point out the relevance of uNKs for stimulating a proper remodeling of spiral arteries. However, IL-15^−/−^ mice neither develop gestational hypertension nor is their progeny growth retarded. This prompts the question whether other cells and/or mediators prevent major consequences in the absence of NKs. The theory of a cell type with similar function and/or secreting comparable mediators at the feto–maternal interface in order to compensate the missing uNKs is interesting and may represent one important mechanism in order to ensure that such a biologically relevant process is fulfilled. We focused here on the analysis of a possible counter-regulation between NKs and MCs at the feto–maternal interface. After finding that MC-deficient animals had increased number of uNKs, we confirmed that similarly, IL-15^−/−^ mice devoid of uNKs have augmented uMCs numbers.

Uterine mast cells are an inhomogeneous population consisting of CTMCs, MMCs, and cells sharing characteristics of both (8). The existence of mixed or unique subpopulations of cells in the uterus is not surprising as it is a tissue with a very high turn over and changes its cellular composition and its functional activity depending on the hormonal influence that defines the cycle stage. Cellular plasticity is, therefore, likely dependent upon the tissue changes occurring in the uterus each cycle and after pregnancy has been established. We also showed that uMCs increased in number after pregnancy establishment and that this was not the case for pregnancies that are characterized by impaired angiogenesis (22). Interestingly, the transfer of regulatory T cells, known to be important for immune balance and immune tolerance in pregnancy, could restore both the number of uMCs and SA remodeling (22). This suggests a tight regulation of the different immune cells to ensure normal pregnancy outcome. It is also likely that cells with the heterogenicity and plasticity of MCs come to the help when an important cell type as uNKs is missing, is diminished in number or affected in its function.

Besides quantifying the number of MCs in the uterus and finding that they were augmented in mice devoid of NKs, we concentrated on parameters that can mirror their activity, as the direct evaluation of their activity in the uterus is technically impaired. In particular, for MC function, their mediators, e.g., tryptases and chymases (23) are relevant. Whereas the chymase Mcpt1 is expressed by MMCs, the tryptase Mcpt6 is especially expressed by CTMCs (24, 25). Here, Mcpt1 and Mcpt6-mRNA levels were analyzed to evaluate MC activity at different pregnancy stages in animals with and without NK cells. Whereas the expression of both proteases was comparable between the groups at gd5, we demonstrated a significant higher expression of Mcpt1 as well as Mcpt6 at gd10, which indicates an increased activity of uMCs at midgestation in IL-15^−/−^ mice. The higher number and activity after gd5 may owe to the fact that they need to secrete factors that otherwise NK cells secrete. In normal pregnant mice, there is a high accumulation and proliferation of uNKs starting from gd4 in the decidua basalis and the MLAp (mesenterial lymphoid aggregate of pregnancy) where they influence important processes like SA remodeling and placentation (26). The significant higher number and increased activity of uMCs could be a mechanism of the body to compensate the missing uNKs in IL-15 deficient mice. The existence of a similar spectrum of mediators of uNKs and uMCs supports this theory. For example, the vascular endothelial growth factor, tumor growth factor-β, angiopoietin-1, IL-8, galectin-1, matrix metalloproteinase (MMP) 2 and MMP9, mediators that are important for angiogenesis or tissue remodeling, are expressed by NKs as well as MCs. IL-1β, IL-6, IL-10, tumor necrosis factor-α, and the granulocyte-macrophage colony-stimulating factor are also expressed by both cell populations (27–29) In our previous study, we surprisingly found that Mcpt5, known as MC mediator, is expressed by uNKs (10). There are a few other examples for cell–cell cooperation to ensure the fulfillment of a relevant biological process. For example, the manipulation of the normal DC/NK ratio leads to a disturbed decidualization and to miscarriage, showing the importance of a well-balanced relationship between cells with similar functions (30). The authors found an unbalanced production of antiangiogenic signals and increased expression of inflammatory genes by an expansion of DCs and simultaneous reduction of NKs; NKs cells seem to counteract the decidual overactivity as a result of a DC expansion (30). Based on our results, we think that a well-regulated activity of mediators expressed by each single cell population is critical for normal pregnancy progression. Brown and colleagues found that also a spatial and temporal regulation of macrophages M1 and M2 polarization is important for several reproductive processes; dysregulation is associated with poor pregnancy outcome (31). While M1 macrophages for example promote Th1 immune responses, M2 macrophages support Th2 immune responses (31). There is an inflammatory milieu around the implantation phase, at which Th1 cytokines are predominant. Th2 cytokines acquire importance later, as they are dominant at mid-pregnancy and contribute to the tolerance state. Changes in the cytokine milieu due to an imbalanced Th1/Th2, and Th17/Treg ratio in the umbilical cord blood can cause preeclampsia (32). Our theory about the redundant roles of uNKs and uMCs is further supported by the fact that simultaneous deletion of both severely compromises fetal well-being by provoking more of half of the progeny to be growth restricted (10). This was proven to be the consequence of nearly un-remodeled spiral arteries and poor angiogenesis in the absence of these two important cell types.

Here, we found that MC-deficient animals had increased number of uNKs and similarly mice devoid of uNKs have augmented uMCs numbers. A limitation of our study is that we do not know at the moment the mechanisms underlying this evident counter-regulation. We are also unable to know at this stage whether the results found in mouse models are transferable and relevant to humans. For both, further studies are needed. In summary, we propose a counter-regulation of uNKs and uMCs as possible compensation mechanism at the feto–maternal interface if one of the cell types is either missing or functional inactive to ensure SA remodeling. Two cell types sharing the important function of supporting the remodeling of the SAs represents a mechanism that ensures the avoidance of fetal distress or growth retardation.

## Ethics Statement

This study was carried out in accordance with the recommendations of the Sachsen Anhalt Ministery (approval number 42502-2-1296UniMD).

## Author Contributions

NM and KW designed, performed, and analyzed experiments. NM substantially contributed to manuscript preparation. MM analyzed data and discussed data. ACZ conceived the studies, supervised the work, and provided financial support. All authors revised the manuscript and were involved in its final approval.

## Conflict of Interest Statement

The authors declare that the research was conducted in the absence of any commercial or financial relationships that could be construed as a potential conflict of interest.
